# Accurate state estimation from uncertain data and models: an application of data assimilation to mathematical models of human brain tumors

**DOI:** 10.1186/1745-6150-6-64

**Published:** 2011-12-21

**Authors:** Eric J Kostelich, Yang Kuang, Joshua M McDaniel, Nina Z Moore, Nikolay L Martirosyan, Mark C Preul

**Affiliations:** 1 School of Mathematical & Statistical Sciences, Arizona State University, Tempe, AZ 85287-1804 USA; 2 Barrow Neurological Institute, St. Joseph's Hospital and Medical Center, 350 W. Thomas Road, Phoenix, AZ 85013 USA

**Keywords:** State estimation, data assimiliation, mathematical models, glioblastoma multiforme

## Abstract

**Background:**

Data assimilation refers to methods for updating the state vector (initial condition) of a complex spatiotemporal model (such as a numerical weather model) by combining new observations with one or more prior forecasts. We consider the potential feasibility of this approach for making short-term (60-day) forecasts of the growth and spread of a malignant brain cancer (glioblastoma multiforme) in individual patient cases, where the observations are synthetic magnetic resonance images of a hypothetical tumor.

**Results:**

We apply a modern state estimation algorithm (the Local Ensemble Transform Kalman Filter), previously developed for numerical weather prediction, to two different mathematical models of glioblastoma, taking into account likely errors in model parameters and measurement uncertainties in magnetic resonance imaging. The filter can accurately shadow the growth of a representative synthetic tumor for 360 days (six 60-day forecast/update cycles) in the presence of a moderate degree of systematic model error and measurement noise.

**Conclusions:**

The mathematical methodology described here may prove useful for other modeling efforts in biology and oncology. An accurate forecast system for glioblastoma may prove useful in clinical settings for treatment planning and patient counseling.

**Reviewers:**

This article was reviewed by Anthony Almudevar, Tomas Radivoyevitch, and Kristin Swanson (nominated by Georg Luebeck).

## 1 Background

Mathematical models, typically a system of ordinary or partial differential equations, can provide considerable insight into the dynamics of biological systems. For initial investigations, it suffices to determine whether a model provides good qualitative agreement with the dynamical process under study. This paper focuses on the issue of quantitative prediction in complex spatiotemporal models of biological processes. In particular, we address the question of how differences between the predicted state of a biological system can be reconciled with noisy measurements to correct the forecast in view of new information; this process is called *data assimilation*. Our overall mathematical approach to data assimilation is quite general and should be broadly applicable to many types of biomathematical models. As an illustration of its potential utility, we consider the possibility of making clinically useful forecasts, in individual patient cases, of the evolution of glioblastoma multiforme (GBM), the most common (and most aggressive) type of human brain cancer. We have chosen GBM because the location and density of the tumor cell population affect patient symptoms and treatment planning, and the dynamics evolve on a complex geometry. However, as we will explain, our data assimilation procedure does not depend on the details of a given cancer growth model and should be broadly applicable to many spatiotemporal models of cancer and other biological phenomena.

**Figure 1:**
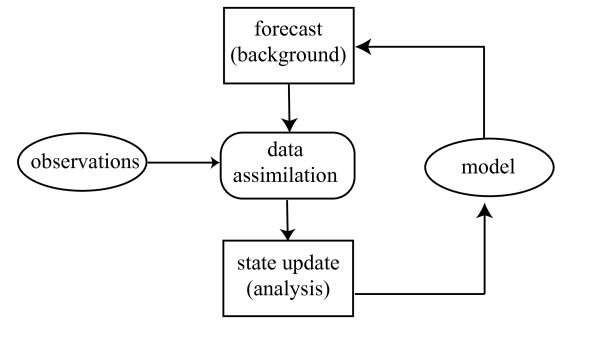
**Schematic illustration of the data assimilation procedure**.

Our approach is derived from one used in numerical weather prediction, illustrated schematically in Figure 1. One begins with a model-generated forecast, often called the *background*. The chaotic evolution of the weather assures that uncertainties in atmospheric initial conditions grow rapidly with time. To make useful predictions, the background must be updated frequently (typically every 6 hours for global models) with noisy (and sometimes sparse) measurements. The data assimilation procedure updates the background in light of the new observations to produce an *analysis*, which, under suitable assumptions, is the maximum likelihood estimate of the model state vector. The model is restarted from the analysis to produce a new background forecast, usually for 6 hours hence in the case of a global weather model. Data assimilation and model forecasts can be combined into *observing system simulation experiments *to quantify the effect of changes in observation accuracy, type, location, and frequency on the accuracy of numerical forecasts. Section 2.3.3 outlines one state-of-the-art procedure for performing the state update in complex spatiotemporal models.

Two significant difficulties must be addressed in the context of GBM. First, many details of the growth of glioblastoma tumor cells are poorly understood, in contrast to the motions of the atmosphere, for which there are well-established physical models. GBM tumors comprise malignant cells with heterogeneous genetic abnormalities and altered metabolism, cysts, cell debris, and vasculature. The patterns by which glioblastomas invade the brain depend on individual growth characteristics and the cytoarchitecture of the surrounding brain tissue.

**Figure 2:**
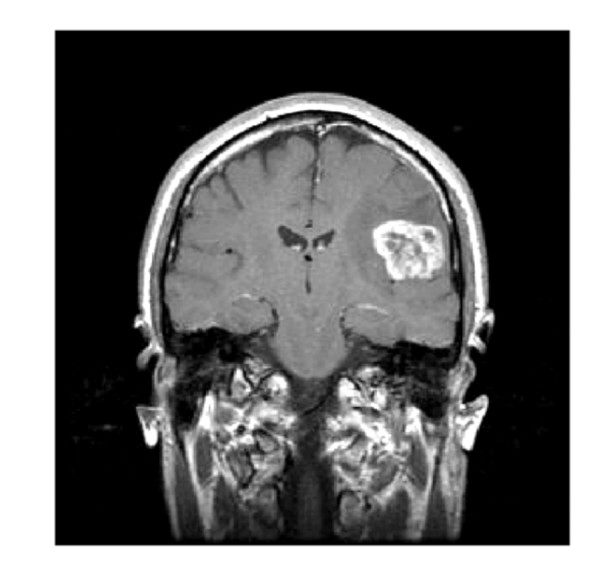
**A representative magnetic resonance image of a GBM patient at initial diagnosis**.

The second problem concerns the interpretation of magnetic resonance (MR) imaging studies. Magnetic resonance imaging, typically performed at intervals of several weeks to months, is the principal means by which the growth and spread of GBM are assessed. Patients are injected with a contrast agent to enhance the visibility of the disruption of the blood-brain barrier. Figure 2 shows a typical MR scan of a patient with a newly diagnosed GBM. The enhancing region (of highest overall intensity) corresponds to the signal from a contrast agent in a dense area of tumor blood vessels. Because these vessels are unusually permeable, the signal probably also reflects contrast agent that has leaked into the surrounding brain tissue. GBM tumors are characterized by profuse abnormal vasculature that is associated with masses of malignant cells, so areas of greatest enhancement are associated with regions of high GBM cell density. Surrounding the central enhancing region is an area of *edema *(swelling) that also may show some contrast enhancement due to tumoral influences on the surrounding brain tissue, which includes abnormal and permeable tumor vasculature and invasion of tumor cells into normal brain tissue [1].

The quantitative relationship between image pixel intensity and tumor cell density is a topic of current investigation. Magnetic resonance images may be manually "segmented" to identify and select those portions of the image that correspond to the actual tumor, edema, etc. Individual variations in brain anatomy, tumor composition, and tumor mass effect also lead to variability in their interpretation, even among expert assessors. Furthermore, variations in contrast uptake, MR signal, and other aspects of image generation may arise from exam to exam. Thus, some ambiguities may occur when mapping a given set of magnetic resonance images to the brain atlas associated with the dynamical model. The interpretation of MR images may be further complicated by treatment: radiation necrosis, for example, may appear similar to new tumor growth [2].

The goal of this paper is to establish that, under reasonable assumptions, good quantitative predictions of GBM growth and spread are possible, as well as estimates of their uncertainty. The discussion is organized as follows. Section 2.1 provides background on GBM tumors and selected mathematical models thereof. Section 2.2 describes the rationale for ensemble forecast methods. Section 2.3.3 outlines a modern data assimilation algorithm called the Local Ensemble Transform Kalman filter. Section 3 describes the results of its application in some observing system simulation experiments, using magnetic resonance images for estimates of the tumor population density with two different models of the growth dynamics, to establish proof of principle of their utility for potential clinical application.

## 2 Methods

### 2.1 Two mathematical models of glioblastoma

Glioblastoma multiforme (GBM) is the most common malignant brain tumor. Despite treatment, patient survival is less than 15 months, on average, from initial diagnosis [3]. GBM tumors are aggressive, largely resistant to chemotherapy and radiotherapy [4], and can quickly invade large and sensitive regions of the brain, making complete surgical resection of the tumor impossible and post-surgical recurrence inevitable [5]. Because little progress has been made against GBM, its biology remains the subject of intense study.

The simulations in this paper involve two mathematical models that attempt to capture the gross dynamics of GBM growth and expansion. Eikenberry *et al*. [6] suggested a model of four stochastic differential equations whose principal dynamics are the diffusive spread and logistic growth of a proliferating and a migrating set of tumor cells. Swanson and co-workers [7,8] considered simpler models of a uniform tumor cell population. In both cases, the models are simulated on a realistic (but static) brain geometry in which the diffusion rates differ between white and gray matter regions.

In the simplest view, the growth of GBM cells is assumed to be exponential, and their spread is governed by Fick's Law, which leads to a model of the form [7]

(1) $$\frac{\partial g}{\partial t}=\nabla \cdot \left(D\left(x\right)\nabla g\right)+\alpha g.$$

The diffusion rate of GBM cells is faster in white matter than in gray matter; often *D *is piecewise constant. The diffusion coefficients, as well as the growth rate *α*, may be approximated from *in vitro *studies, sequential MR studies of individual patients, or the Einstein-Stokes relation [7].

Equation 1 predicts that the tumor cell density can become unbounded. A potentially more realistic model is Gompertzian or logistic growth to some local carrying capacity *T*_max_; in the latter case, the model becomes [9]

(2) $$\frac{\partial g}{\partial t}=\nabla \cdot \left(D\left(x\right)\nabla g\right)+\alpha g\left(1-\frac{g}{T_{\max}}\right).$$

Typical values for the parameters in Eq. (2), which we will call the *logistic Swanson *model, are reported in Table 1. Another model, by Eikenberry *et al*. [6], divides the cancer cell population into proliferating and migrating classes and also attempts to capture the degradation of the extracellular matrix by the invading tumor. In this paper, we consider a simplified version of the Eikenberry model, which assumes that there is a net transition of cells from the proliferating to the migrating phenotype along the tumor front, gradually degrading the extracellular matrix (ECM).

**Table 1:** Representative parameters for the logistic Swanson model, Eq.(2), in two dimensions.

Location-independent parameters	Meaning	value		
*α*	maximum glioma growth rate	0.2 day^-1^		
*T*_max_	glioma carrying capacity	10 000 cells mm^-2^		
**Location-dependent parameters**	**Meaning**	**White Matter**	**Gray Matter**	**CSF**

*D*(**x**)	diffusion rate (mm^2 ^day^-1^)	0.0065	0.0013	0.001

The net growth of the proliferating cells is logistic (this term also incorporates the net transition from the migrating to the proliferating phenotype as well as cell death due to crowding). The dependent variables are

$$\begin{array}{c} \;g\left(x,\;t\right)\;=\;\text{proliferating}\;\text{cell}\;\text{density}\\ \;m\text{(}x\text{,}\;t\text{)}\;=\;\text{migrating}\;\text{cell}\;\text{density}\\ \;w\left(x,\;t\right)\;=\;\text{extracellular}\;\text{matrix}\;\left(\text{ECM}\right)\;\text{density} \end{array}$$

and the two-phenotype model is expressed as a coupled set of three partial differential equations, as follows.

(3) $$\begin{array}{c} \frac{\partial g}{\partial t}\;=\;\underset{\text{diffusion}}{\underset{︸}{\nabla \cdot \left(D_{G}\left(x\right)\nabla g\right)}}+\alpha g\underset{\text{logistic}\;\text{growth}}{\underset{︸}{\left(1-\frac{g+m}{T_{\max}}\right)}}\\ \;\;-\underset{\text{directed}\;\text{migration}\;\text{into}\;\text{ECM}}{\underset{︸}{\nabla \cdot \left(\chi \left(x\right)g\nabla w\right)}} \end{array}$$

(4) $$\frac{\partial m}{\partial t}\;=\;\underset{\text{diffusion}}{\underset{︸}{\nabla \cdot \left(D_{M}\left(x\right)\nabla m\right)}}+\underset{\text{directed}\;\text{migration}\;\text{into}\;\text{ECM}}{\underset{︸}{\nabla \cdot \left(\chi \left(x\right)g\nabla w\right)}}$$

(5) $$\frac{\partial w}{\partial t}\;=\;\underset{\text{degradation}}{\underset{︸}{-\rho w\left(\frac{g+m}{\theta_{W}+g+m}\right)}}+\underset{\text{repair}}{\underset{︸}{\alpha_{W}w\left(1-w\right)}}$$

Table 2 displays the nominal parameter values for the two-phenotype model, Eqs. (3)-(5). The values used here differ slightly from those in [6] and were chosen so that the total tumor cell populations from both the logistic Swanson model, Eq. (2), and the two-phenotype model grow at approximately the same rate.

**Table 2:** Nominal values of the parameters for the two-phenotype model, Eqs.(3)-(5), in two dimensions.

Parameter	Meaning		value	
α	maximum glioma growth rate		0.025 day^-1^	
*T*_max_	glioma carrying capacity		10 000 cells mm^-2^	
*α_W_*	maximum ECM recovery rate		0.01 day^-1^	
*ρ*	maximum ECM remodeling rate		0.02 day^-1^	
*θ_W_*	cell density at half-maximum ECM degradation		100 cells mm^-2^	
**Parameter**	**Meaning**	**White Matter**	**White Matter**	**CSF**

*D_G_*(**x**)	growing cell diffusion rate (mm^2 ^day^-1^)	0.002	0.0004	0.001
*D_M_*(**x**)	migrating cell diffusion rate (mm^2 ^day^-1^)	0.10	0.02	0.001
*χ*(**x**)	haptotaxis coefficient (mm^-1^)	0.25	0.05	0

Both sets of equations are integrated using a brain geometry from the BrainWeb database, developed by the McConnell Brain Imaging Center of the Montreal Neurological Institute at McGill University [10]. We use the discrete anatomical model of a normal brain generated for McGill's MR simulator, which consists of a 181 × 217 × 181 isotropic grid of 1 mm^3 ^voxels in Talairach space [11]. Each voxel is classified as background, cerebro-spinal fluid (CSF), gray or white matter, fat, muscle/skin, skin, skull, or glial matter. To reduce the computational expense, the equations are integrated over a representative coronal slice at the center of the 3-dimensional domain, from which voxels representing the skull and other non-brain tissue have been removed. The resulting 2-dimensional domain is a fixed 145 × 143 grid (the mass effect is not modeled). For simulation purposes, glial matter is treated as white matter, and the diffusion coefficients (*D_G _*and *D_M_*, as appropriate) are piecewise constant.

The spatial derivatives are approximated by finite differences, and the resulting set of ordinary differential equations is integrated over the 2-dimensional coronal domain using the second-order (in time) Heun's method with a fixed time step (0.1 day^-1^). Given the discrete nature of the brain geometry, location-dependent parameters (such as the diffusion constants) are taken to be piecewise constant.

[Although a forward integration method for finite difference schemes can be unstable, the authors believe that Heun's method provides a reasonable compromise between numerical stability and simplicity of implementation for testing the state estimation procedure described here. The robustness of the integration scheme has been tested by halving, doubling, and quadrupling the nominal domain resolution. In all cases, the 90-day tumor population, integrated from a fixed initial cell distribution, varied by less than 10 percent for suitably small time steps (typically 0.05-0.5 day), which was judged satisfactory for our purposes here. Implicit solvers require significant effort to implement because the brain geometry induces complicated no-flux boundary conditions; nevertheless, implicit solvers may be required for choices of model parameters that make the equations stiff.]

**Figure 3:**
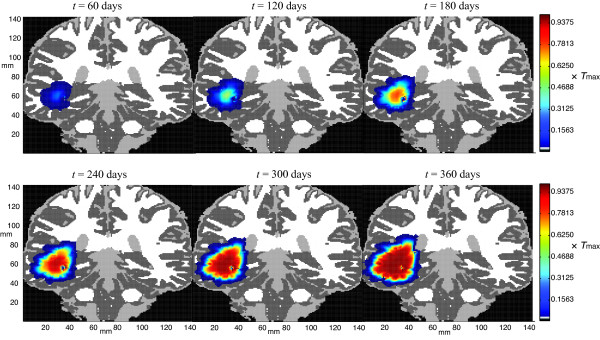
**The evolution of a typical GBM tumor under the two-phenotype model**. The tumor cell density is color-coded as a fraction of the local carrying capacity for this representative solution of Eqs. (3)-(5) for the nominal parameter values given in Table 2.

Figure 3 shows the evolution of a typical GBM tumor under the two-phenotype model, Eqs. (3)-(5), for the nominal parameter values given in Table 2. The initial condition is prepared by integrating a population of 100 growing and 10 migrating cells in a single 1 mm^2 ^voxel for 365 days, which under these parameters yields a starting population of approximately 10^5 ^cells covering about 150 mm^2^. The equations are integrated over the indicated 2-dimensional coronal slice for an additional 360 days; snapshots of the tumor cell density at 60-day intervals are plotted in Figure 3. (The axes show the spatial extent of the domain in millimeters.)

The bar on the right shows the color coding of cell density: dark blue (lowest density) to dark red (highest density). More precisely, the cell population density is mapped to one of 128 "bins," each of which corresponds to a given color. The darkest blue color corresponds to voxels in which the tumor cell density is between $\frac{3}{128}T_{\max}$ and $\frac{4}{128}T_{\max}$, and so on to the darkest red color where the cell density approaches *T*_max_. The brain domain is shown wherever the tumor cell density falls below $\frac{3}{128}T_{\max}$; this color coding is dark gray for gray matter, white for white matter, and light gray for CSF. We presume that the warmer colors correspond approximately to the enhancing region in an MR scan and cooler colors to a portion of the visible edema; tumor cells are present at a nontrivial density $\left(\text{up to}\frac{3}{128}T_{\max}\right)$ in a region extending 2-4 mm beyond the periphery of the blue-shaded area.

We have chosen the logistic Swanson and two-phenotype models because they are adequate to establish the potential feasibility of a data assimilation (state estimation) scheme in the face of significant errors in model parameters and data acquisition. One must integrate several dozen different initial conditions and parameters in parallel, which can be done in a reasonable period on a multicore laptop computer for these particular models. Both models give plausible simulations of the natural history of a GBM tumor from initiation to diagnosis, but the omission of mass effect is a limitation, and we do not wish to suggest that one provides a better mathematical representation of GBM biology than the other. Interested readers may consult [12] for a survey of mathematical models of glioma.

### 2.2 Ensemble forecasting

In a classic 1963 paper [13], Edward Lorenz showed that a simple model of fluid flow, consisting of three coupled ordinary differential equations, exhibits what is now called chaotic behavior. Such a system is sensitive to small changes in initial conditions: simulations started from states that initially are close together quickly diverge. Although trajectories from typical initial conditions (i.e., those that are not fixed points or unstable periodic orbits) appear to approach the same limit set, they become uncorrelated after awhile even when the initial conditions are close together. The implications for weather forecasting are clear: the atmosphere cannot be sampled everywhere, all observations are noisy, and no forecast model is perfect. These factors, with the chaotic dynamics, imply that there is a finite time horizon past which weather forecasts are no more accurate than climatological averages.

Even on time scales of a few days or less, uncertainties in the initial state of the atmosphere may lead to substantial forecast errors. In a 1965 paper [14], Lorenz suggested that, instead of running one forecast from a best guess of the initial state, one should run an *ensemble *of many forecasts, each from a statistically equivalent estimate of the initial state, to give a Monte Carlo estimate of the forecast uncertainty for a given weather model. Under appropriate assumptions, the ensemble mean becomes an empirical maximum-likelihood forecast. By 1992, supercomputers had become sufficiently powerful to make ensemble forecasting a practical part of the daily operations at the U.S. and European weather centers [15].

**Figure 4:**
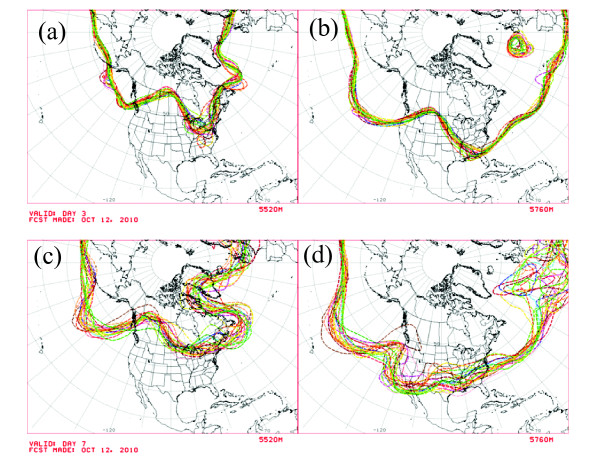
**Representative "spaghetti plots" of ensemble forecasts**. Shown are contours of the 500 hPa geopotential height over North America for forecasts started on Oct. 12, 2010. (a)-(b) Predicted values after 3 days for the 5520-m and 5760-m contours, respectively. (c)-(d) Predicted values after 7 days for the 5520-m and 5760-m contours, respectively.

Figure 4 shows representative ensemble forecasts of geopotential height contours at 500 hPa (about half of the mean surface pressure). Each curve shows the result, from one initial condition on Oct. 12, 2010, of a forecast obtained by running the weather model for 3 days (top panels) and 7 days (bottom panels). Roughly speaking, the maps show the predicted locations where half the atmosphere's mass is below 5520 m (left panels) and 5760 m (right panels). (The geopotential, Φ(*z*), is the work needed to raise a unit mass a vertical distance *z *from mean sea level and accounts for the variation of the earth's gravitational field with latitude and elevation. The geopotential height is Φ(*z*)/*g*_0_, where *g*_0 _= 9.80665 m s^-2 ^is the global average of gravitational acceleration at mean sea level. For more details, see Chapter 1 of [16].) Of greatest interest here is the forecast uncertainty, which varies considerably in space as well as in time. Because of the chaotic dynamics, the forecast uncertainty generally is larger at 7 days than at 3 days. The 5760-m contours (right panels) show considerable spread over the North Atlantic Ocean at 7 days, corresponding to especially large uncertainties in the forecast of the 500-hPa geopotential height.

Unless the initial conditions are updated sufficiently often, numerical weather models produce forecasts that are only as accurate as an almanac's. Modern operational meteorology relies on state estimation procedures that are based on the Kalman filter, described in Section 2.3.1. The Kalman filter in turn relies on an accurate characterization of the forecast uncertainty, i.e., the covariance matrix associated with the model state vector. Depending on the resolution, a contemporary weather model may have on the order of 10^6 ^to 10^10 ^components in its state vector. The associated covariance matrix is far too large to be stored on a supercomputer, even if one were able to estimate all the elements. Methods to reduce the dimensionality of the estimation problem therefore are essential. A forecast ensemble can provide an empirical, low-rank approximation of the forecast covariance matrix, and spatial localization restricts the scope of the analysis to regions where the forecast dynamics are most highly correlated. (For example, during the 6-hour interval over which weather models are updated, atmospheric conditions over New York and San Francisco are effectively independent.)

The ensemble approach can be adapted to the cancer models, Eq. (2) and Eqs. (3)-(5). Although the logistic terms do not foster chaotic dynamics, the forecast uncertainty increases with time due to errors in the initial tumor population and in the model parameters. In addition, the dimensionality problem remains: at 1 mm resolution, the spatial domain for the human brain contains more than 1 million grid points.

The results presented in Sec. 3 are obtained from an ensemble of 50 model realizations of an underlying "true" tumor, i.e., a tumor whose dynamics are given exactly by Eqs. (3)-(5) with the parameter values in Table 2. For each realization, the growth rate *α *and carrying capacity *T*_max _are chosen from uniform distributions centered about the nominal values in Tables 1 and 2. (Once fixed, they remain constant for the duration of the simulation; Table 3 shows the range of each distribution.) In addition, each realization uses a different estimate of the initial tumor density within each grid box (see Sec. 3). The tumor model is integrated to produce a 60-day forecast of the state of the tumor. At that time, we imagine that a new MR image becomes available that provides a noisy observation of the tumor cell population. The Local Ensemble Transform Kalman Filter, described next, updates the forecast ensemble using the MR data. The updated ensemble is used to create a subsequent 60-day forecast, and so on. The process stops if it diverges or if the tumor grows so large as to be fatal.

**Table 3:** Parameter intervals for the forecast model, Eq.(2), used to integrate the ensemble solutions in the observing system simulation experiments.

Experiment 1	Experiment 2	Experiment 3
0.01767 ≤ *α *≤ 0.035347	0.0153 ≤ *α *≤ 0.0612	0.0153 ≤ *α *≤ 0.10
(260 to 520 days)	(150 to 600 days)	(90 to 600 days)
8000 ≤ *T*_max _≤ 12000	8000 ≤ *T*_max _≤ 12000	8000 ≤ *T*_max _≤ 12000
2.0 × 10^-3 ^≤ *D_w _*≤ 2.0 × 10^-2^	2.0 × 10^-4 ^≤ *D_w _*≤ 2.0 × 10^-2^	2.0 × 10^-4 ^≤ *D_w _*≤ 2.0 × 10^-1^

### 2.3 Data assimilation

In this section, we briefly describe the rationale and algorithmic implementation of the Local Ensemble Transform Kalman Filter (LETKF) for data assimilation. (See Hunt *et al*. [17] and Ott *et al*. [18] for a detailed mathematical justification.) The basic problem may be stated informally as follows: Given a forecast model consisting of a coupled system of ordinary differential equations, $\dot{u}=F\left(u,\;t\right)$, find the trajectory **u**(*t*) that best fits the observations. In the case of meteorology, the dynamical system **F **is deterministic, but there is uncertainty in the initial condition, **u**(*t*_0_). (More generally, one can regard **F **as having a stochastic component.) Suppose that, for *i *= 1, 2, . . . , *n *- 1, we have a vector of observations **y***_i _*that is related to the system state by **y***_i _*= **H***_i_*(**u**(*t_i_*)) + *ε_i_*, where *ε_i _*is a Gaussian random variable with mean **0 **and covariance matrix **R***_i_*. In the scenario envisioned here, the *observation operator ***H***_i_*(**u**(*t_i_*)) is the MR image that, given a perfect model **F **in the absence of noise, would result from a tumor whose density in each grid box is **u**(*t_i_*) = **u***_i_*. Data assimilation is an application of weighted least squares, as we now describe.

#### 2.3.1 The Kalman filter

We motivate our approach by first considering the case of a linear model, **u***_i _*= **M***_i_***u**_*i*-1_, whose observations are a linear combination of the system state: **y***_i _*= **H***_i_***u***_i _*+ *ε_i_*. (We follow the development in [17] here.) A maximum-likelihood approach suggests that the "most likely" trajectory {**u***_i_*} is one that minimizes the quadratic *cost function*

(6) $$\overset{n-1}{\underset{i=1}{\sum }}{\left(y_{i}-H_{i}u_{i}\right)}^{\text{T}}R_{i}^{-1}\left(y_{i}-H_{i}u_{i}\right).$$

The Kalman filter provides an iterative method to compute the minimizer. Suppose that, at time *t*_*n*-1_, we have a minimizer ${\bar{u}}_{a_{n-1}}={\bar{u}}_{a}\left(t_{n-1}\right)$ with an associated covariance matrix $P_{a_{n-1}}$, that is,

(7) $$\overset{n-1}{\underset{i=1}{\sum }}{\left(y_{i}-H_{i}u_{i}\right)}^{\text{T}}R_{i}^{-1}\left(y_{i}-H_{i}u_{i}\right)={\left(u-{\bar{u}}_{a_{n-1}}\right)}^{\text{T}}P_{a_{n-1}}^{-1}\left(u-{\bar{u}}_{a_{n-1}}\right).$$

One can regard ${\bar{u}}_{a_{n-1}}$ and $P_{a_{n-1}}$ as the mean and covariance, respectively, of a Gaussian probability distribution that represents the relative likelihood of the possible system states given the observations at *t*_1_, . . . , *t*_*n*-1_.

Absent further information, the most likely estimate of the system state at *t_n _*is the model forecast,

(8) $$u_{b_{n}}=M_{n}u_{a_{n-1}}.$$

Its associated covariance matrix is

(9) $$P_{b_{n}}=M_{n}P_{a_{n-1}}M_{n}^{\text{T}}+C_{n}.$$

Under a linear model, a Gaussian distribution of states at time *t*_*n*-1 _propagates to a Gaussian distribution at *t_n_*. Model errors increase the uncertainty, which can be approximated by taking **C***_n _*as a symmetric positive definite matrix.

If a new observation vector **y***_n _*becomes available at *t_n_*, then it can be shown [17] that the relation (7) is satisfied if the updated state estimate ${\bar{u}}_{a_{n}}$ minimizes

(10) $$J\left(u\right)={\left(u-{\bar{u}}_{b_{n}}\right)}^{\text{T}}P_{b_{n}}^{-1}\left(u-{\bar{u}}_{b_{n}}\right)+{\left(y_{n}-H_{n}u\right)}^{\text{T}}R_{n}^{-1}\left(y_{n}-H_{n}u\right).$$

Equation (11) weights the forecast and the observations. roughly speaking, the minimizer is closer to the quantity with the smaller covariance. The minimizer is

(11) $${\bar{u}}_{a_{n}}={\bar{u}}_{b_{n}}+P_{a_{n}}H_{n}^{\text{T}}R_{n}^{-1}\left(y_{n}-H_{n}{\bar{u}}_{b_{n}}\right)$$

where

(12) $$P_{a_{n}}={\left(I+P_{b_{n}}H_{n}^{\text{T}}R_{n}^{-1}H_{n}\right)}^{-1}P_{b_{n}}.$$

The matrix $P_{a_{n}}H_{n}^{\text{T}}R_{n}^{-1}$, often called the *Kalman gain *matrix, describes how to apportion the discrepancies between the actual and predicted observations to yield the increment between the forecast ("background") state, ${\bar{u}}_{b_{n}}$, and its update ("analysis"), ${\bar{u}}_{a_{n}}$.

Equation (11) shows that it is possible to compute updated maximum-likelihood estimates of *all *components of the model state vector, even if they cannot all be measured, provided that the observations are reasonably correlated with the model state. For example, suppose a Kalman filter is applied to the two-phenotype model, Eqs. (3)-(5), where the state vector **u **contains components (*g*, *m*, *w*), corresponding to the growing and migrating cell densities, plus the relative density of the ECM, at each point of the domain. Also suppose that it is possible to make noisy measurements only of the total GBM cell density at each grid point. The observation operator, **H**(**u**), would then be the predicted value, *g *+ *m*, of the total GBM cell density at each grid point. Equation (11) shows how to ascribe the difference between the predicted and observed values of total cell density to *each *component, (*g*, *m*, *w*), in the update of the grid point in question (and Eq. (12) estimates their covariance), even though the densities of the growing and migrating cells cannot be measured separately.

#### 2.3.2 Variations on the Kalman filter

As mentioned in Section 2.2, one difficulty with a naive application of the Kalman filter is that the covariance matrices of the background and analysis states, $P_{b_{n}}$ and $P_{a_{n}}$ respectively, are very large. In addition, the models that we are considering are nonlinear, which implies that the background (forecast) covariance matrix $P_{b_{n}}$ cannot be computed as a simple matrix product.

There are three overall approaches to the latter problem. One is the *extended *Kalman filter, which attempts to estimate $P_{b_{n}}$ through a suitable integration of a linearized model (i.e., the associated variational equations) [19]. The principal difficulty with this approach is that it is highly dependent on the model equations. It is difficult to linearize a large model, and if the model equations change, then so does their linearization. Data assimilation systems based on this approach are tightly coupled to the forecast model.

A second approach is the *unscented *Kalman filter, in which so-called "sigma points" are chosen about the ensemble mean and integrated with the model to estimate the forecast covariance matrix [20]. The unscented Kalman filter relies on adequate sampling of the error probability distribution, which becomes impractical once the dimension of the model state space is sufficiently large.

The third approach is an application of the Monte Carlo method: run an ensemble of forecasts, as described in Sec. 2.2, to find a low-rank approximation of the forecast covariance matrix $P_{b_{n}}$. If one can find suitable sets of initial conditions from which to integrate the model, then the corresponding forecasts can be used to parametrize (at least approximately) the distribution of the forecast error [19]. The ensemble approach is *model independent *insofar as it does not rely explicitly on the model equations; rather, $P_{b_{n}}$ is estimated empirically from the forecast state vectors.

The ensemble must be large enough to provide an adequate sample of the space of forecast uncertainties. With sufficient sampling, the unscented and ensemble filters should yield the same results as the extended filter. However, the model linearization may be difficult to program, and the integration of the variational equations is computationally expensive. The Local Ensemble Transform Kalman Filter, described next, is an ensemble method. Although it is not a fundamentally new approach to state estimation, extensive tests with complex atmospheric models have shown that it is computationally efficient, easily parallelizable, and highly accurate [21,22].

#### 2.3.3 The Local Ensemble Transform Kalman Filter

When the model (or observation operator) is nonlinear, Eqs. (10)-(12) must be modified. The background (forecast) covariance matrix $P_{b_{n}}$ is no longer a simple matrix product and must be approximated by other means, as described in Sec. 2.3.2. In addition, the (suitably modified) cost function *J *may have no unique minimizer, and even if one exists, there is no guarantee of optimality, in the sense of being an unbiased estimator with minimum variance. Nevertheless, schemes that seek to minimize cost functions similar to Eq. (10) have proven useful in operational meteorology (see [15] and references therein for an extensive bibliography).

The objective of an ensemble scheme is to choose a set of analysis vectors whose spread about ${\bar{u}}_{a_{n}}$ provides a suitable approximation of the state uncertainty $P_{b_{n}}$. Computational limitations generally restrict the number of ensemble members, *k*, to be less than a few hundred--much less than the number of state variables in most cases. Nevertheless, if the background ensemble suitably approximates $P_{b_{n}}$, then it is possible to generate an accurate analysis *without knowing the model equations explicitly*. This aspect makes the LETKF (like other ensemble Kalman filters) a model-independent data assimilation system. An update of the form (12) accounts for forecast uncertainties only in the *k*-dimensional subspace spanned by the ensemble. If the underlying dynamical process has more than *k *positive Lyapunov exponents, then an analysis of the form (11) cannot correct forecast errors outside the span of the ensemble subspace.

The LETKF, therefore, is applicable to models that exhibit *local low dimensionality*--that is, models whose local dynamics over short time intervals can be regarded as relatively low dimensional but driven by the dynamics of neighboring regions [23]. Experience suggests that many geophysical models exhibit this property. The logistic growth term in the GBM models considered here also leads to local low dimensionality: once an initial population of cells invades a given volume of the brain, it grows to an asymptotic value. The region of greatest uncertainty in any GBM forecast is the location of the tumor "front," as the rate at which GBM cells diffuse into healthy tissue may vary significantly with time and location [24].

The idea behind the LETKF is to perform a local analysis that requires the ensemble to capture the forecast uncertainty in only a portion of the state space. Each local analysis involves a separate linear combination of the ensemble solutions over a given local region. In this way, the dimensionality of the global analysis is much larger than *k*. Extensive investigations have shown that the LETKF is an accurate and computationally efficient data assimilation system for complex geophysical models, including the Global Forecast System, which is the U. S. Weather Service's operational model [22]; a coastal estuarine model of New York Harbor [25]; and a dynamical model of the Martian atmosphere [26], among others.

**Figure 5:**
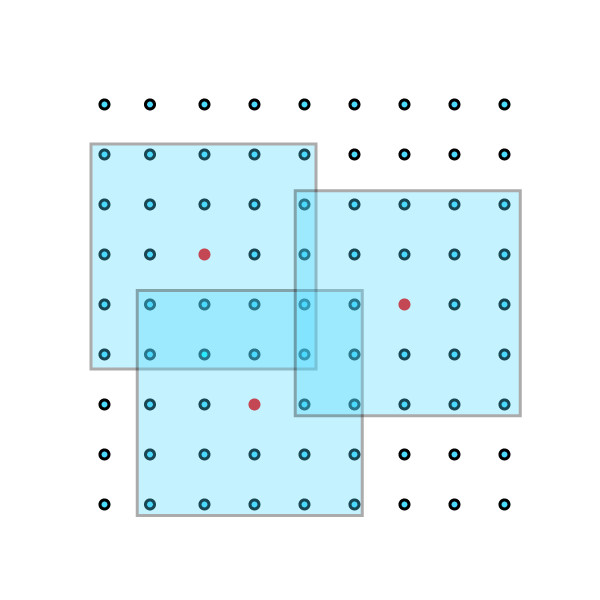
**Schematic illustration of the LETKF localization procedure**. Observations in each local region (shown in blue) are used to update the grid point in the center (shown in red).

We briefly outline the implementation of the LETKF used to obtain the results in Sec. 3. The overall objective is to use the observations contained within a suitable local region to update the state estimate of the grid point in the center. In other words, the LETKF finds the minimizer of Eq. (10) one grid point at a time within the subspace spanned by the ensemble solutions. (The "cookbook" below provides a step-by-step outline.) Figure 5 illustrates the idea schematically for local regions consisting of 5 × 5 grid boxes. In each case, the grid point in the center of the local region (marked in red) is updated using observations located anywhere under the pale blue cover. Because the local regions belonging to adjacent grid points overlap considerably, the set of observations used to update the grid points tends to vary relatively slowly as a function of location, assuming that the observations are sufficiently dense. This property helps to assure the continuity of the analysis, as explained below. Although the mathematics does not require that the local regions be squares or circles, or even that they be centered exactly on the grid points, it is convenient to define them as such in actual implementations, except possibly near the boundaries of the model domain. For simplicity of exposition, we refer to the "center" as the grid point being updated by observations in the local region. Each grid point is updated independently, so the computations may be performed in parallel; in this way, the LETKF may be implemented efficiently on modern computers.

The following discussion summarizes the considerations and computational procedure that attend to each local region. The global analysis is computed grid point by grid point, using suitable local regions around each. The size of the local regions may be fixed (as in the results reported here) or may vary by location.

##### Spatial localization

As noted above, the dynamics in a selected local region often may be regarded as low dimensional (either chaotic or stochastic) and driven by the dynamics of neighboring regions. In the case of a global weather model, a local region is about 1000 km × 1000 km, which is approximately the spatial extent of a typical high-or low-pressure system in the midlatitudes. Insofar as operational weather models are updated four times daily, this choice roughly corresponds to the atmospheric region that has the greatest impact on the weather at a given point during a typical 6-hour period. Modern atmospheric observing networks are sufficiently dense that updates for adjacent grid points in regions of this size use most of the same observations, which fosters continuity in the analysis. The LETKF is relatively insensitive to choices of ensemble and local region size, provided that both are within a reasonable range. For this initial GBM study, the local regions are 7 mm × 7 mm squares. The region coincides with the computational grid (which has 1-mm spacing). Our choice of 7 × 7 grids comes from an empirical assessment that the areas of greatest forecast uncertainty are along and near the edges of the tumor core, that is, near the boundary of the region with highest contrast on the MR scan (cf. Figure 2). In the situation described here, the local region size should be comparable to the spatial correlation length of the tumor dynamics; since the tumor "front" is of greatest interest, local regions from 5 mm × 5 mm to 11 mm × 11 mm should suffice. We have used ensemble sizes of 25 and 50 in our simulations with roughly comparable results. Larger ensembles tend to provide better parametrizations of the distribution of forecast uncertainties; the results described in Sec. 3 have been computed with 50-member ensembles.

##### Ensemble

We assume that, at time *t_n_*, a set of background ensemble forecasts, $u_{b_{n}}^{i}$, *i *= 1, 2, . . . , *k *is available. Each $u_{b_{n}}^{i}$ is a vector containing the full set of model variables over the entire domain. We denote by $x_{b}^{i}$ the components of $u_{b_{n}}^{i}$ associated with the model grid point at the center of the local region. (In Sec. 2.1, we used **x **to denote a given spatial location within the domain of the PDE models. Here $x_{b}^{i}$ denotes the model state at a particular location. In the case of the two-phenotype model, Eqs. (3)-(5), $x_{b}^{i}$ is the 3-vector (*g*, *m*, *w*) giving the density of proliferating and migrating cells and the extracellular matrix at the grid point in question.)

Suppose that the solution vector at each model grid point contains *m *components (e.g., *m *= 3 in the case of the two-phenotype model) and that there are *ℓ *observations in the local region. Compute the mean, ${\bar{x}}_{b}$, of the ensemble state components $x_{b}^{i}$, *i *= 1, 2, . . . , *k*, and the *m *× *k *ensemble perturbation matrix **X***_b _*whose *i*th column is $x_{b}^{i}-{\bar{x}}_{b}$.

The LETKF seeks to minimize an objective function ffo the form (10) *within the subspace spanned by the forecast ensemble*. In other words, rather than finding an estimate of the entire state vector **x**, we seek a linear combination of the ensemble forecasts that minimizes Eq. (10) for the components of **x **that correspond to a given local region within the physical grid of the model and that lie in the ensemble subspace. As a consequence, the minimizer has the form $x={\bar{x}}_{b}+x_{b}w$, and the "cookbook" below shows how to calculate **w**.

One important consideration is that the columns of **X***_b_*, by construction, sum to **0 **and therefore do not form a basis for the subspace spanned by the ensemble solutions. In particular, the *k*-vector whose components are 1 belongs to the null space of **X***_b_*, so the rank of the *k *× *k *ensemble covariance matrix $P_{b}={\left(k-1\right)}^{-1}x_{b}x_{b}^{\text{T}}$ is at most *k *- 1. However, **X***_b _*is one-to-one on its column space *S*, so we regard **X***_b _*as a linear transformation from an abstract *k*-dimensional space $\tilde{S}$ to *S *and minimize *J *on *S*, relative to which **P***_b _*has a well defined inverse. It can be shown that if $w\in \tilde{S}$ is Gaussian with mean **0 **and covariance matrix (*k *- 1)^-1^**I**, then $x={\bar{x}}_{b}+x_{b}w$ is Gaussian with mean ${\bar{x}}_{b}$ and covariance matrix **P***_b _*[17].

##### Observations and data selection

The observation operator **H **need not be linear. Only the components within the local region are selected for the analysis. Let $h_{b}^{i}$ denote the *ℓ *vector of the components of the observation operator $H\left(u_{b_{n}}^{i}\right)$ within the local region. Let **y***_n _*be the (global) vector of observations. As with **H**, only the components of the observation vector **y***_n _*that belong to the local region (Figure 5) are used; denote them by **y***_o_*. As with the model state vectors, we let ${\bar{y}}_{b}$ be the mean of the vectors $h_{b}^{i}$, *i *= 1, 2, . . . , *k *and define the *ℓ *× *k *matrix **Y***_b _*whose *i*th column is $h_{b}^{i}-{\bar{y}}_{b}$. In what follows, we also assume that the observation error covariance matrix **R **has been truncated to the observations within the local region.

We assume that **H**, if it is nonlinear, can be approximated as $H\left({\bar{x}}_{b}+x_{b}w\right)\approx {\bar{y}}_{b}+y_{b}w$. The goal is to find a linear combination, **w**, of the ensemble solutions to minimize the cost function

(13) $$J^{*}\left(w\right)=\left(k-1\right)w^{\text{T}}w+{\left[y_{o}-{\bar{y}}_{b}-y_{b}w\right]}^{\text{T}}R^{-1}\left[y_{o}-{\bar{y}}_{b}-y_{b}w\right],$$

which is the analogue of Eq. (10) in the subspace spanned by the spatially localized ensemble solutions [17]. The first term, (*k *- 1)**w**^T^**w**, represents the forecast uncertainty and has a particularly simple form by virtue of the representation of the ensemble subspace in terms of the perturbation vectors that form **X***_b_*.

The remaining steps are a "cookbook" recipe for computing **w **and the local analysis ensemble.

1. Compute the *k *× *ℓ *matrix $C=y_{b}^{\text{T}}R^{-1}$. (If the observations are not independent and **R **is not diagonal, it is computationally more efficient to solve the system **RC**^T ^= **Y***_b _*instead of inverting **R**.)

2. Compute the *k *× *k *symmetric matrix ${\tilde{P}}_{a}={\left[\left(k-1\right)I∕\rho +\;Cy_{b}\right]}^{-1}$. (See below for more discussion of *ρ*.)

3. Compute the *k *× *k *matrix ${\tilde{w}}_{a}={\left[\left(k-1\right){\tilde{P}}_{a}\right]}^{1∕2}$, by which we mean the symmetric square root. This choice ensures that ${\tilde{w}}_{a}$ depends continuously on the elements of ${\tilde{P}}_{a}$. (Otherwise, small changes in ${\tilde{P}}_{a}$ at neighboring grid points can lead to very different analysis ensembles [17,27].)

4. Compute the *k*-vector ${\bar{w}}_{a}={\bar{P}}_{a}C\left(y_{0}-{\bar{y}}_{b}\right)$ and add it to each column of ${\tilde{w}}_{a}$ to form the *k *× *k *analysis weight matrix **W***_a_*.

5. Compute the analysis perturbation matrix **X***_a _*= **X***_b_***W***_a_*.

6. The analysis ensemble, $x_{a}^{i}$, is formed by adding ${\bar{x}}_{b}$ to the *i*th column of **X***_a_*, *i *= 1, 2, . . . , *k*.

##### Global analysis ensemble

The global analysis ensemble, $u_{a_{n}}^{i}$, consists of the collection of local analysis ensembles, $x_{a}^{i}$, at the center of each local region.

##### Covariance inflation

In principle, the only free parameters in the LETKF scheme are the ensemble size, *k*, and the size of each local region. In practice, however, the model is not a perfect representation of the underlying dynamics. As a result, ensemble methods tend to underestimate the actual background uncertainty, which causes them to underweight the observations in the analysis scheme. In severe cases, the filter can diverge. One *ad hoc *remedy is to "inflate" the background ensemble covariance by a tunable parameter. The procedure described above has the effect of multiplying the background ensemble perturbations by $\sqrt{\rho }$.

### 2.4 Observing system simulation experiments

In meteorology, tests of proposed data assimilation systems are called *observing system simulation experiments*. Because the weather is a complex multiscale process, one hopes to separate the effects of observation density, location, and error from model error. In a *perfect model *simulation, one creates a "truth run" from a fixed initial condition with the same model that is used to make the ensemble forecasts. At intervals, synthetic noisy observations are generated from the "truth." The goal of the simulation experiment is to determine how well a forecast ensemble tracks the truth when the synthetic observations are assimilated using a forecast model that is identical to the model used for the truth run [21]. Such experiments can quantify the effect of noise and observation density and frequency on the accuracy of the analyses, since there is no model error. (The assimilation of actual atmospheric observations, of course, provides a test of the data assimilation system in the presence of model error. Since the truth is not known, the analysis quality is assessed using a surrogate, such as the root-mean-square difference between a 48-hour forecast started from the ensemble mean and the corresponding observations.)

In contrast to the usual situation in meteorology, where most of the governing equations of the atmosphere are well established, the forecast models considered here are relatively crude approximations of the underlying dynamics. GBM tumors comprise a heterogeneous population of cells, and, although the tumor as a whole may grow and spread at rates that are reasonably well described by the nominal parameter values, mutations among the genetically unstable population may cause the growth and migration rates to change unpredictably from their nominal values.

Furthermore, in a clinical setting, every patient receives treatment (usually some combination of surgery, radiation, and chemotherapy), whose effects have not been well characterized in the mathematical models described here. For these reasons, we use different models to generate the observations and the forecasts in the results described below.

#### 2.4.1 Forecast model and ensemble generation

Given the current state of knowledge, errors in any contemporary forecast model for GBM are likely to be significant, and we wish to establish the efficacy of the data assimilation scheme under such circumstances. For the observing system experiments described here, we take as the "truth" a tumor whose growth dynamics are supposed to be governed exactly by the two-phenotype model, Eqs. (3)-(5), with the parameter values given in Table 2. Synthetic observations of the true tumor consist of noisy MR images whose overall intensity is assumed to vary linearly with cell density. They are assimilated at regularly spaced intervals to update an ensemble of initial conditions for which the forecast model is Eq. (2), the logistic Swanson model. A similar model has been used to assess the survival times in individual GBM patients following surgical resection [9], and it can be integrated readily for many different sets of initial conditions on a laptop computer. (We could just as well have used the logistic Swanson model for the "truth" tumor and the two-phenotype model as the forecast model. Qualitatively similar results would obtain, but the computational expense would be considerably greater.)

The filtering scheme described in Sec. 2.3.3 is applied to a 50-member forecast ensemble once every 60 days, and the simulation is continued for 360 days to assess its accuracy and stability. This process is necessarily limited in duration, because the tumor eventually grows to a size that causes fatal complications. No attempt has been made to assess the effect of treatment, which is a subject for future investigation.

Our principal focus is the effect of model and observation uncertainties on the effectiveness of our data assimilation approach. To attempt to capture the heterogeneity of GBM tumors, we consider an ensemble of models: each ensemble solution is integrated using Eq. (2) with a unique set of parameter values as well as initial conditions. In the results described here, we choose random values within certain intervals of the logistic growth rate *α*, carrying capacity *T*_max_, and the diffusion rate *D *in white matter, which remain fixed for the duration of the simulation (see Table 3). Alternatively, one might allow the parameters to vary with time, possibly according to a random process with drift, but this simple setup suffices to demonstrate the viability of the overall approach.

#### 2.4.2 Generation of synthetic observations

The operator **H**(**x**) gives the quantity that would be observed if the tumor state vector were **x**. As discussed in the introduction, many details of the relationship between tumor cell density and contrast enhancement are not well characterized, and there is intrinsic variability in contrast agent uptake and other aspects of MR image generation. Hence we assume that **H **has a random component. For our purposes here, **H**(**x**) represents the contrast enhancement (above a baseline level) due to the presence of tumor cells and that the enhancement varies linearly with the tumor cell density at each point of the domain, plus a random value.

The value of **H **is computed pointwise as follows. Let *u_k_*(**x**, *t*) be the tumor cell density for the *k*th ensemble member at location **x **at time *t*. Let

(14) $$h_{k}\left(x\right)=\max\left(0,\min\left(1,\frac{u_{k}\left(x,t\right)}{T_{\max}^{k}}+\eta \left(x\right)\right)\right),$$

where *η*(**x**) is a uniformly distributed random value in [-0.1, 0.1] and $T_{\max}^{k}$ is the carrying capacity for the *k*th ensemble solution. The value of *h_k_*, which is clamped to the unit interval, is the component of **H **corresponding to location **x **in the brain domain. (The *η*'s are independent.)

Equation (14) represents an idealized situation, because it ignores the mass effect of the tumor and assumes that there is a one-to-one mapping between pixels in the generated observation and grid points in the model domain. A mathematical characterization of contrast enhancement in individual clinical cases, as well as the registration errors in the mapping between the model domain and MR image, are subjects of ongoing investigation.

#### 2.4.3 Data assimilation and analysis procedure

Each observing system simulation experiment proceeds as follows. Steps 1 and 2 constitute the initialization phase.

1. The "truth tumor" is integrated according to the two-phenotype model, Eqs. (3)-(5), with the parameter values given in Table 2, to produce the sequence of states shown in Figure 3, which are then used to generate all the observations as described above.

2. An initial ensemble of 50 solutions of the logistic Swanson model, Eq. (2), is prepared by choosing an initial cell density randomly and uniformly from the interval [50,150] in a single voxel within 3 mm of that used to start the truth tumor. Each ensemble solution has a unique set of model parameters that are chosen randomly and uniformly from the intervals given in Table 3; they remain constant for the duration of the simulation. Each single-voxel "seed" is integrated for 365 days and produces an initial tumor of about 10^5 ^to 10^6 ^cells. Three sets of observing system simulation experiments are performed, using parameters chosen from the intervals listed in the respective columns of Table 3.

3. After the truth and ensemble solutions are prepared as described in Steps 1 and 2, the reanalysis phase begins. We assimilate a synthetic MR image that has been generated from the truth tumor according to Eq. (14) and the Local Ensemble Transform Kalman Filter is applied as described in Sec. 2.3.3 using a 7 mm × 7 mm local region and a modest covariance inflation factor (*ρ *= 0.1). The updated ("analyzed") ensemble solutions are integrated for 60 days to produce a new background forecast.

4. Step 3 is repeated at *t *= 60, 120, 180, 240, 300, and 360 days, for a total of seven assimilation steps and six forecast cycles.

Three such experiments are conducted with forecast model parameters chosen randomly and uniformly from the intervals in Table 3 for the logistic Swanson model, Eq. (2). In the case of purely logistic growth, *g*' = *αg*(1 - *g*/*T*_max_), one can solve explicitly to find the value of *α *for which the time needed for *g *to increase from 1 percent to 99 percent of *T*_max _equals a specified value. The first two lines of Table 3 report those values; for example, in Experiment 1, the smaller *α *yields an interval of approximately 520 days for the tumor cell density to increase from 0.01*T*_max _to 0.99*T*_max _and the larger value, about 260 days. The quantity *D_w _*refers to the value of the diffusion coefficient *D*(**x**) in white matter. We take *D*(**x**) to be piecewise constant, and its value in gray matter is fixed at the nominal value in Table 1. (GBM cells tend to migrate along white matter tracts [28-30] and the two-dimensional domain chosen for these simulations contains considerably more white matter than gray matter.)

Both mathematical models considered in this paper predict that the cell density at every point in the core of a GBM tumor eventually reaches the same constant value, *T*_max_. Such a situation is biologically suspect (as Figure 2 suggests) and also causes problems for ensemble Kalman filtering schemes: if all tumors reach the same density everywhere, then the background covariance matrix approaches zero in local regions in and near the tumor core. Consequently, the first term in the objective function, Eq. (10), tends to infinity and the filter gives no weight to the observations; this situation leads to the eventual divergence of the filter. In the simulations here, we let *T*_max _be a random parameter that is fixed for each ensemble solution. Alternatively, one can let *T*_max _vary randomly in space. Both choices prevent the background covariance matrix from becoming too ill-conditioned.

## 3 Results

**Figure 6:**
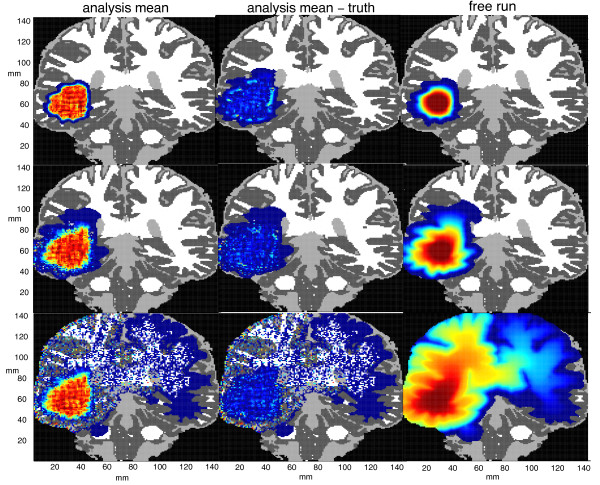
**Results of the observing system simulation experiments after the final assimilation step**. The first, second, and third rows show the results of Experiments 1, 2, and 3, respectively, at *t *= 360 days using the parameter ranges listed in the respective columns of Table 3. The left column shows the final ensemble analysis mean, and the middle column, the pointwise absolute difference between the analysis mean and the "true" tumor. The right column shows the ensemble mean of free runs of the models, i.e., the mean 360-day forecast without data assimilation.

The goal of the observing system simulation experiments here is to shadow the "true" tumor, shown in Figure 3, using synthetic observations and a forecast ensemble as described in Sec. 2.4. Figure 6 shows the results of three assimilation experiments following the final assimilation step at *t *= 360 days. The first, second, and third rows correspond, respectively, to Experiments 1, 2, and 3 in Table 3. The left column, labeled "analysis mean," shows the ensemble mean after the final analysis step, 360 days after initialization; it is the pointwise average of the fraction of the carrying capacity over all the ensemble members. (The color coding is the same as in Figure 3.) The right column, labeled "free run," shows the corresponding ensemble means after 360 days when no data assimilation is performed. The middle column shows the pointwise absolute difference between the total cell population in the analysis mean and in the true tumor. At most points, the numerical value of this pointwise difference is generally a few percent of *T*_max_, so it is colored dark to light blue.

Figure 6 shows that the performance of the data assimilation system degrades gracefully as the extent of parameter misspecification increases. Even in the worst case (Experiment 3), where the white-matter diffusion rate varies by three orders of magnitude and the logistic growth rate by more than a factor of six in the forecast model, the final analysis provides a reasonably good approximation of the core of the "true" tumor (shown at the bottom right of Figure 3). Although the accuracy of the forecasts in Experiment 3 is considerably poorer than in Experiments 1 and 2, the analysis is reasonably good, but it demonstrates considerable uncertainty regarding the spatial extent of the lowest-density cell distribution.

**Figure 7:**
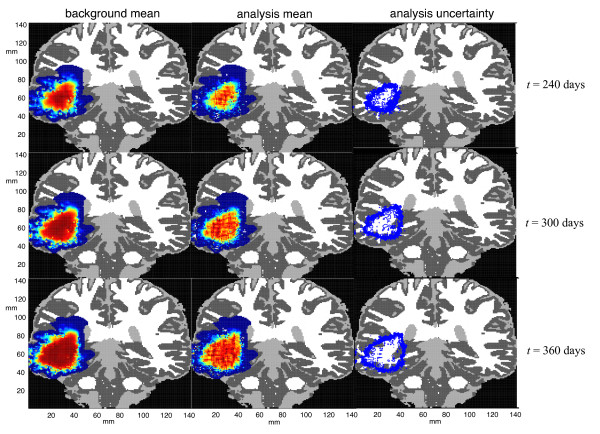
**Example of forecast correction**. The paneels show how the LETKF data assimilation algorithm corrects the background forecasts in Experiment 2 at *t *= 240, 300, 360 days. The left column shows the mean of the 60-day background forecast mean for the indicated period and the middle column shows the analysis mean after the synthetic noisy MR imaging data have been assimilated. The right column shows a "spaghetti plot" showing the contour, for each of the 50 ensemble members, where the total tumor cell density is one-half of its maximum value.

Figure 7 shows the background forecasts during the last three cycles of Experiment 2 and their corresponding analyses at *t *= 240, 300, and 360 days, respectively. The left column shows the mean of the forecast ensemble, which is a 60-day prediction started from the previous analysis ensemble. (The color coding, which is as in Figure 3, shows the pointwise mean of the tumor cell density at each point, averaged over the 50 ensembles.) The middle column shows the analysis mean, i.e., the corrected background forecast ensemble after the synthetic data are assimilated at the indicated time. The third column is a "spaghetti plot" showing, for each ensemble solution, a contour plot of where the tumor cell density is one-half the carrying capacity, i.e., $\frac{1}{2}T_{\max}$. These contours span a 5-6 mm margin, which gives an indication of the uncertainty in the boundary of the highest cell density. The forecast extent of lowest cell density has a greater span, because we have assumed that the noise in our synthetic MR scans, generated according to Eq. (14), is larger on a proportional basis in low-density regions. This assumption reflects our belief that the boundaries of edematous regions are harder to resolve than those of the tumor core.

Comparable results, not shown here, are obtained when the two-phenotype model, Eqs. (3)-(5), is used as the ensemble forecast model. In this situation, other key parameters, such as the haptotaxis coefficient *χ*(**x**) and the migrating cell diffusion coefficient *D_M_*(**x**), are chosen from intervals of varying width. The results are also relatively insensitive to the size of the ensemble (for example, an ensemble of size 25 works almost as well) and to the size of the local region (e.g., 5 mm × 5 mm to 11 mm × 11 mm local regions yield approximately similar results).

## 4 Discussion

This preliminary study demonstrates the potential feasibility of ensemble forecasting and data assimilation methods for short-term prediction of the growth and spread of malignant brain tumors. Our principal focus is on the efficacy of a Kalman-type filter for estimating initial conditions from noisy imaging data. Although the immediate application is to glioblastoma, the design and implementation of the Local Ensemble Transform Kalman Filter (Sec. 2.3.3) do not depend on the particular equations of a given mathematical model. Hence, this forecasting and state update approach may prove useful in other biomathematical investigations.

Unlike the case in meteorology, there are no first-principles models for the dynamics of glioblastoma. Consequently, model error is likely to be a significant confounding factor in any state estimation scheme for GBM and similar diseases. We have attempted to simulate the effect of model error by using one model of GBM growth, Eqs. (3)-(5), to generate a "truth tumor" and another, Eq. (2), for the forecast and update cycle. We chose Eq. (2) for this purpose because of its elegance and simplicity and because it has been shown to provide useful predictions of patient survival in clinical cases [9]. Our state estimation approach, the Local Ensemble Transform Kalman Filter (LETKF), appears to be robust and stable, at least for time periods of clinical relevance, even in the presence of considerable error in model parameters, therefore, we believe that the LETKF warrants careful consideration in future efforts to synthesize mathematical models and clinical data for predictive purposes in individual patient cases.

Nevertheless, considerable work remains before our approach can be seriously considered in clinical settings. Many challenges are common to all mathematical simulations of cancer [31] and to glioma in particular [32,33]. We outline a few of them here.

### The mathematical models

The preliminary investigation here makes no attempt to account for the effects of treatment. The parametrization of any mathematical model of treatment must account for many variables, including the timing and dosage of radiation [12,34], chemotherapy [35], systemic steroids [36], and mass effect [37-39]. *Model error*. Mathematical forecast models of glioblastoma (and other cancers) are likely to suffer significant errors, which are treated only crudely in the simulations described here. Improved mathematical characterizations of forecast model error, including model parameter calibration and more accurate quantification of uncertainties in the state estimate and its covariance in the presence of systematic errors, is a topic of ongoing research [40-42].

### Magnetic resonance imaging

The correspondence (if any) between tumor cell density and contrast enhancement in MR images needs to be established. One must assess the variability in operational settings for a clinical scan (including but not limited to magnet strength, pulse sequencing, and the dosage of contrast agent) and the variability among patients (for example, the rate of uptake and metabolism of contrast agent). Although a statistical predictor of glioma grading based on MR imaging has been proposed [43], the authors are unaware of any studies that attempt to relate cell density to contrast enhancement in MR images.

### Image registration

Besides the problem of determining the initial density of tumor cells, one needs a geometrical atlas for the model. This can be done using a standard set of such atlases, such as the BrainWeb database [10], or one can attempt to construct an atlas from each individual patient. There is considerable variability even between the brains of healthy people. For example, the brains of men and women differ, on average, in gross total volume and in the distribution of gray and white matter [44]. The mass effect of GBM tumors adds to the difficulty. The registration error must be accounted for in the observation covariance matrices used in the data assimilation procedure.

### Non-Gaussianity of data

Finally, to simplify the mathematics, ensemble Kalman filtering schemes assume that the errors in the data and the model are gaussian (or can be adequately approximated by gaussian distributions). The previous considerations may result in error statistics that deviate significantly from gaussianity. Future work should characterize the error statistics in clinical cases and adapt the minimization strategies in the LETKF accordingly.

## 5 Conclusions

The Local Ensemble Transform Kalman Filter provides an accurate and computationally efficient way to update the state vector (initial condition) of a complex spatiotemporal model with new quantiative measurements. Its efficacy relies only on the local low dimensionality of the underlying model dynamics, not on the equations themselves, and so provides a flexible state update scheme even as the models themselves are improved. An accurate forecast system for glioblastoma may prove useful in clinical settings for treatment planning and patient counseling. The model independence of the LETKF provides a flexible framework for other mathematical modeling efforts in biology and oncology.

## List of abbreviations used

**CSF: **cerebrospinal fluid; **ECM: **extracellular matrix; **GBM: **glioblastoma multiforme; **LETKF: **Local Ensemble Transform Kalman Filter; **MR: **magnetic resonance,

## Competing interests

The authors declare that they have no competing interests.

## Authors' contributions

EJK planned the research and implemented the parallel computations on which the reported results are based. YK directed the development and parametrization of the two-phenotype mathematical model. JM carried out initial simulations of the mathematical models and the data assimilation algorithm. NZM and NLM made substantial contributions to the acquisition, analysis, and interpretation of magnetic resonance image data of previous patient cases and assisted with the literature review. MCP contributed to the project conception and design, conducted much of the literature review, and revised and reviewed the sections on glioblastoma biology. All authors read and approved the final manuscript.

## Authors' information

EJK is Professor of Mathematics at Arizona State University. His current research includes data assimilation in weather and climate models as part of the Mathematics and Climate Research Network, funded by the National Science Foundation (DMS-0940314).

YK is Professor of Mathematics at Arizona State University. His research interests include mathematical models of tumor growth and management, as well as stiochiometry-based population models and their implications. He is an editor-in-chief of the journal *Mathematical Biosciences and Engineering*.

JM is a Ph.D. candidate in applied mathematics in the School of Mathematical and Statistical Sciences at Arizona State University.

NZM and NLM are neurosurgery fellows at the Barrow Neurological Institute.

MCP holds the Newsome Chair for Neurosurgery Research and directs the Neurosurgery Research Laboratory at the Barrow Neurological Institute in Phoenix, AZ.

## Reviewers' comments

The authors sincerely thank the reviewers for their careful reading of the manuscript and their suggestions for improvement. In the reports reproduced below, we have replaced references to page numbers in the review manuscript with section numbers and have omitted comments about typographical errors, all of which we have corrected.

### Reviewer's report 1

*Tomas Radivoyevitch, Case Western Reserve University, USA*.

This paper is important because the approach presented is generally applicable, and because the notion that states can be observed (i.e. estimated/inferred) even if they cannot be directly measured, needs to receive more attention in biology. This is a very well written paper.

Major compulsory revisions:

One thing the paper could use is a little more clarification [in Sec. 2.3] regarding how the LETKF is model independent. Specifically, is it that the Kalman gain in Eq. (10) has been replaced by a tuned asymptotic observer matrix that is now merely tuned for algorithm convergence kinetics and thus independent of the model? Or, in the simple case of a linear model, is it that the background state **u***_b _*is somehow no longer $Mu_{b_{n-1}}$ i.e., somehow now independent of **M**? It needs to be made clear whether "model independence" means everything is 100% data driven, or whether it means that all possible underlying nonlinear models are reduced to linear models, so it matters not matter what the true underlying nonlinear model is (in which case one might argue that the method depends on the linear model that it is reduced to, and thus is not model independent). In the paragraph just before Sec. 2.3, regarding uniform distributions centered about nominal values in Tables 1 and 2, please state the range (lower and upper limits) of the uniform distribution used. This should also be done just before Sec. 2.4.2.

*Authors' response: *We have attempted to clarify this point by adding a new subsection (Sec. 2.3.2 in this version of the paper), which motivates the various approaches to Kalman filtering for nonlinear models. The LETKF, like all ensemble approaches, does not rely on a statically tuned model covariance matrix. Instead, the background covariance matrix, $P_{b_{n}}$, is estimated empirically from the forecast ensemble. Equally importantly, the LETKF also estimates the covariance of the updated state vector in light of the new observations at each step. The variational equations of the model are not needed, and in this respect, the LETKF is a model-independent approach. Our methodology requires that the background and analysis perturbations provide a reasonable local linearization of the dynamical model and observation operator, as described in the discussion in Sec. 2.3.3 leading to Eq. (13). We have added references to Table 3, which provides the range of parameter values used in the simulations, at the appropriate points in Sec. 2.2 and Sec. 2.4.1.

### Reviewer's report 2

*Kristin Swanson, University of Washington, USA (nominated by Georg Luebeck, Fred Hutchinson Cancer Research Center, USA)*.

This paper illustrates how one might use an established method of data assimilation, the Local Ensemble Transform Kalman Filter, to update the state vector of a system given new data when modeling glioblastomas. This is done by presenting two different models for glioblastoma, using one to generate a "truth" with which to update the predictions of the other. Since synthetic data is used, this is clearly a proof of concept and there are many pitfalls this method may incur when attempting to apply this technique clinically. The authors do mention at least some of these. In the field of glioblastoma modeling this is certainly a new technique and worth considering. Though, its power would be increased if combined with a technique for patient specific model calibration as well. In general, the paper is well written and presented. There are just a few comments and concerns we have that should be addressed.

Comments:

1. While it is not the goal of the paper to assess assumptions of the models used, it should be noted that there is actually a neglible amount of extracellular matrix in the brain.

*Authors' response: *Although the brain contains little physically static tissue matrix compared to the other organs in the body, there is still an extracellular matrix that mediates the behavior of cells within the brain and becomes active in states of disease. For example, the brain ECM tends to resist invasion by metastatic tumors [45]. The disruption of the brain ECM occurs in various neurodegenerative diseases [46] and appears to be reorganized in GBM tumors [47]. In any case, the model (3)-(5) makes no assumption about a particular physical or chemical form of the brain ECM, which is still in the early stages of characterization. The model simply assumes that there is a generalized barrier that is degraded near the tumor front and promotes invasion of tumor cells. We do not assert that the two-phenotype model is a "better" model of GBM than the one-phenotype model; it is used merely as a proxy for a "true" tumor whose internal dynamics are more complex than those represented in a forecast model.

Concerns:

1. Section 2.1: In the vast majority of the work done by Swanson *et al*., the value of *D *in the CSF is taken to be 0. Admittedly, this is not mentioned in the 2003 paper or in the 2008 paper, but neither is the value of 0.001 listed. It is not physical for the tumor to grow relative to which **P***_B _*has a well defined inverse. doubtfully change the proof of concept presented, it should be remarked upon and kept in mind for future use.

*Authors' response: *We appreciate this clarification. Although tumor cells do not proliferate in the CSF, it seems probable that they diffuse into the CSF at a nonzero rate, hence, a small value for *D*(**x**) seemed more plausible to us than a no-flux condition. We agree that the precise value of *D*(**x**) within the CSF, as long as it is small, is not likely to significantly affect the dynamics of either model considered here.

2. According to Table 2 and Table 3, the values of *D_g_*, *D_m_*, and *D *in the CSF regions are all the same value. Thus, the comment in the paragraph introducing the two-phenotype model regarding their relative values seems incorrect.

*Authors' response: *As mentioned above, we chose small values of these coefficients to reflect a nonzero rate of diffusion into the CSF. The rates are identical for both cell phenotypes because, for the moment, we have no reason to believe that they should be significantly different. In both models, the cell diffusion rates are taken to be greater in white matter than in gray matter.

3. In the last paragraph of Sec. 2.3.1 it is said that it is shown in Sec. 3 that "it is possible to estimate the densities of both the growing and migrating cell populations. . ." However, in Sec. 3 it is only mentioned that it can be done, but never shown. This should either be added as an additional full example or the comment should be modified.

*Authors' response: *We have added a paragraph of explanation regarding this point at the end of Sec. 2.3.1.

4. Figure 5 would better illustrate the method of localization if a third box were added with the center grid point within one of the other regions. That is, it would better illustrate how every grid point is associated with its own local region if it was illustrated that the "primary" point can be within another local region.

*Authors' response: *We thank Dr. Swanson for this suggestion for improvement, which has been incorporated into Figure 5 (and its caption).

5. In the Spatial Localization paragraph of Sec. 2.3.2 [now Sec. 2.3.3], it is mentioned LETKF is relatively insensitive to ensemble and local region size provided they are within a reasonable range. Please provide the approximate ranges you tested to give more intuition as to just how insensitive they are.

*Authors' response: *We have included more details on this point in the discussion in Sec. 2.3.3, which replaces Sec. 2.3.2 in the original manuscript.

6. In the Ensemble paragraph of Sec. 2.3.2 [now Sec. 2.3.3], an example is given for $x_{b}^{i}$ as a 4 vector including the a variable for chemorepellent. Such a variable was never introduced in Eqs. (3)-(5). Also, this is inconsistent with the next sentence saying, e.g., *m *= 3. It seems the variable *c *should be removed.

*Authors' response: *We have made this correction.

7. In the observation and data paragraph of Section 2.3.2 more intuition should be given to the first term of the objective function. It is likely a regularization, but an explicit description should be provided. Also, more intuition for what the "cookbook" is doing would be good. It seems like it should be finding a zero of the derivative of the objective function, but the steps do not give an immediate feel for that.

*Authors' response: *We thank Dr. Swanson for this helpful suggestion and have added a few paragraphs of explanation about this matter in Sec. 2.3.3.

8. Regarding the comments in the final paragraph before the results section about *T*_max_. The situation that the cell density is uniform within the core of the tumor is indeed biologically suspect. But taking *T*_max _as spatially variable or as a random parameter does not seem to be the best way to combat this. In fact, those solutions also seem biologically suspect since it indicates the maximum cells that can occupy a region. A better solution would be to include cell death in the model and allow for a necrotic core (what is actually seen in Figure 2).

*Authors' response: *GBM tumors are a heterogeneous group of neoplasms, not all of which have a necrotic core. The mottled appearance in Figure 2 may reflect differential uptake of contrast agent within the tumor vasculature, areas of cysts and hemorrhage, and regions of viable as well as necrotic tissue. The distinguishing characteristic of glioblastoma tumors upon microscopic examination is multiple necrotic foci surrounded by so-called pseudopalisading cells [48]. Neither mathematical model discussed here captures this behavior.

9. Figure 6. An improvement to the image would be to add an additional column showing the difference between the "truth" and the analysis mean in some way, perhaps by showing the 0.5*T*_max _contours from each on the same graphic. This would help in your claim of accuracy, as now you are appealing to the readers extremely rough "eye-ball" norm for saying the mean (shown in one figure) is accurate against the truth (shown in another figure).

*Authors' response: *We have revised Figure 6 so that the central column shows the absolute pointwise difference between the total cell densities between the "true" tumor and the analysis mean. The color coding is on the same scale as the other columns.

10. Regarding the discussion. There have been many attempts at models of various treatment modalities and these should be mentioned.

*Authors' response: *We have revised the discussion and included additional references on this topic.

11. Again regarding the discussion. Why should a patient always be registered to an atlas? Ultimately, that would take away from "patient-specific" information. Why not generate meshes from the individual patient's images? Of course, these images would need to be registered to each other, but it does not seem that computation on the atlas geometry would be or should be considered optimal.

*Authors' response: *These questions will be the focus of future research efforts.

12. It might be informative to include a small discussion of how this differs from parameter calibration and could be complemented with parameter calibration: i.e., as the parameters will vary drastically from patient to patient, to reduce the uncertainty in the prediction, a calibration would be useful to reduce the range of values the LETKF would sample from.

*Authors' response: *Parameter calibration is an essential part of model tuning and improvement. As far as the LETKF is concerned, the distinction between model parameters and initial conditions is arbitrary: one can augment the state vector with components that represent the model parameters and estimate the augmented vector using the LETKF [40]. We have not done so in this investigation, because (among other things) the models we consider do not capture the effects of treatment, which may select for different subpopulations of tumor cells, affect the patient's immune response, and alter the dynamics of the original tumor.

### Reviewer's report 3

*Anthony Almudevar, University of Rochester, USA*.

The authors apply Kalman filter methodology to the problem of spatio-temporal modeling of brain cancer growth based on sequences of MRI images. A number of well-known models are considered, the one selected for demonstration models assumes logistic tumor growth (there are one- and two- phenotype models involving proliferating cell density, or proliferating and migrating cell density). A main theme of the article is an analogy with weather forecasting models, and an adaption of methodology successfully used in that field to the current problem. As has been well established, such forecasting models are very sensitive to small perturbations of initial conditions (i.e., are chaotic). One technique for stabilizing predictions is to take an average over models using slightly varying initial states and parameters. This procedure, coupled with data assimilation (updating initial conditions with new data) is incorporated into what is referred to in the literature as the local ensemble transform Kalman filter (LETKF). The paper is interesting, well motivated and very well written. The models and application are clearly described with sufficient detail, and I believe would be of interest to readers of *Biology Direct*. I have three concerns.

The authors point out that the cancer growth model "does not foster chaotic dynamics" (Section 2.2). This being the case, I think it would be important to discuss whether any other technique would accomplish the same goals set out in the article. The ensemble method seems to resemble a computational Bayesian approach, which might be naturally defined given the underlying statistical model. The authors might consider a brief section in which alternative approaches are compared. It would also be good to summarize in the same section how the problem is characterized by the theory of dynamic systems or numerical analysis, that is, why techniques associated with chaotic systems are needed. These points are raised at various places in the paper, but it might be better to have a single subsection summarizing the justification for this choice.

*Authors' response: *Dr. Almudevar's points are well taken, and we have added a new section, 2.3.2, that attempts to provide a brief outline of some possible approaches to state estimation without greatly lengthening the present paper. In addition, we have amplified the discussion of local low dimensionality in Sec. 2.3.3 to explain why the efficacy of the LETKF can be expected in the context considered here.

The methodology is tested on data simulated from a specified model, assumed perfectly known (Table 2). Three implementations of LETKF are applied, differentiated by the ensemble definitions. Although one is noticeably less accurate, all three are viable, and reasonably consistent (Figure 6). The predictions are compared to a "free run" (column 3, Figure 6), computed without the data-assimilation component, but still using ensemble means. Here, there is considerably more variation in the predictions. Thus, the efficacy of the data-assimilation but not the ensemble-mean component of the method is demonstrated.

In the numerical demonstration, the true [tumor] is generated using the two-phenotype model, but the one-phenotype model is used in the forecast. The authors write "(We could just as well have used the logistic Swanson model [one-phenotype model] for the 'truth' tumor and the two-phenotype model as the forecast model. Qualitatively similar results would obtain, but the computational expense would be considerably greater)" [Sec. 2.4.1], and later write "Comparable results, not shown here, are obtained when the two-phenotype model, Eqs. (3)-(5), is used as the ensemble forecast model" [end of Sec. 3]. What is the rationale for not using the same model as true and forecast model, say, the one- and two- phenotype model demonstrated separately?

*Authors' response: *It is perfectly reasonable to use the same model to generate both the forecasts and the synthetic observations, particularly when testing a data assimilation system. The first author did just this in the context of the Global Forecast System weather model (Ref. [21] provides more details and rationale). Although we have not reported the results here, the LETKF gives excellent agreement between the true and shadowed tumors when the same model is used for both observations and forecasts. However, such a result does not demonstrate the potential utility of a data assimilation system in the context of cancer, where model error is likely to be substantial. This is our motivation for using two different models. No choice of forecast model parameters can exactly match the "true" tumor, but the data assimilation system with one-phenotype model, Eq. (2), nevertheless provides good forecasts in the presence of a moderate degree of error and uncertainty in the model parameters.

## References

[B1] Gossman A Helbich TH Kuriyama N Ostrowitzki S Roberts TP Shames DM van Bruggen N Wendland MF Israel MA Brasch RC Dynamic contrast-enhanced magnetic resonance imaging as a surrogate marker of tumor response to anti-angiogenic therapy in a xenograft model of glioblastoma multiforme J Magn Reson Imaging 2002 15 233 240 10.1002/jmri.10072 11891967

[B2] Rijpkema M Kaanders JH Joosten FB van der Kogel AJ Heerschap A Method for quantitative mapping of dynamic MRI contrast agent uptake in human tumors J Magn Reson Imaging 2002 14 457 463 11599071 10.1002/jmri.1207

[B3] Norden AD Wen PY Glioma therapy in adults Neurologist 2006 12;279 292 10.1097/01.nrl.0000250928.26044.47 17122724

[B4] Amberger VR Hensel T Ogata N Schwab ME Spreading and migration of human glioma and rat C6 cells on central nervous system myelin in vitro is correlated with tumor malignancy and involves a metalloproteolytic activity Cancer Res 1998 58 149 158 9426071

[B5] Demuth T Berens ME Molecular mechanisms of glioma cell migration and invasion J Neurooncol 2004 70 217 228 10.1007/s11060-004-2751-6 15674479

[B6] Eikenberry SE Sankar T Preul MC Kostelich EJ Thalhauser CJ Kuang Y Virtual glioblastoma: growth, migration and treatment in a three-dimensional mathematical model Cell Prolif 2009 42 511 528 10.1111/j.1365-2184.2009.00613.x 19489983 PMC6760820

[B7] Swanson KR Alvord EC Jr Murray JD A quantitative model of differential motility of gliomas in white and grey matter Cell Prolif 2000 33 317 329 10.1046/j.1365-2184.2000.00177.x 11063134 PMC6621920

[B8] Swanson KR C B Murray JD Alvord EC Jr Virtual and real brain tumors: using mathematical modeling to quantify glioma growth and invasion J Neurol Sci 2003 216 1 10 10.1016/j.jns.2003.06.001 14607296

[B9] Swanson KR Rostomily RC Alvord EC Jr A mathematical modeling tool for predicting survival of individual patients following resection of glioblastoma: A proof of principle Brit J Cancer 2008 98 113 119 10.1038/sj.bjc.6604125 18059395 PMC2359692

[B10] BrainWeb: Simulated Brain Database http://www.bic.mni.mcgill.ca/brainweb

[B11] Talairach J Tournoux P Co-Planar Stereotaxic Atlas of the Human Brain: 3-D Proportional System: An Approach to Cerebral Imaging 1988 New York: Thieme Medical Publishers

[B12] Harpold HLP Alvord EC Jr R SK Evolution of mathematical modeling of glioma proliferation and invasion J Neuropathol Exp Neurol 2007 66 1 9 10.1097/nen.0b013e31802d9000 17204931

[B13] Lorenz EN Deterministic non-periodic flow J Atmos Sci 1963 20 130 141 10.1175/1520-0469(1963)020<0130:DNF>2.0.CO;2

[B14] Lorenz EN A study of the predictability of a 28-variable atmospheric model Tellus 1965 17 321 333 10.1111/j.2153-3490.1965.tb01424.x

[B15] Kalnay E Atmospheric Modeling, Data Assimilation, and Predictability 2003 Cambridge, UK: Cambridge University Press

[B16] Horton JR An Introduction to Dynamic Meteorology, 4th ed 2004 Amsterdam: Elsevier Academic Press

[B17] Hunt BR Kostelich EJ Szunyogh I Efficient data assimilation for spatiotemporal chaos: A local ensemble transform Kalman filter Physica D 2007 230 112 126 10.1016/j.physd.2006.11.008

[B18] Ott E Hunt BR Szunyogh I Zimin AV Kostelich EJ Corazza M Kalnay E Patil DJ Yorke JA A local ensemble Kalman filter for atmospheric data assimilation Tellus A 2004 56 415 428 10.1111/j.1600-0870.2004.00076.x

[B19] Evensen G Data Assimilation: The Ensemble Kalman Filter 2007 Berlin: Springer-Verlag

[B20] Julier SJ Uhlmann JK Durrant-Whyte HF A new approach for filtering nonlinear systems Proc of the American Control Conference 1995 3 1628 1632 IEEE

[B21] Szunyogh I Kostelich EJ Gyarmati G Patil DJ Hunt BR Kalnay E Ott E Yorke JA Assessing a local ensemble Kalman filter: Perfect model experiments with the NCEP global model Tellus A 2005 57 528 545 10.1111/j.1600-0870.2005.00136.x

[B22] Szunyogh I Kostelich EJ Gyarmati G Kalnay E Hunt BR Ott E Satterfield E Yorke JA A local ensemble Kalman filter data assimilation system for the NCEP global model Tellus A 2008 60 113 130

[B23] Patil DJ Hunt BR Kalnay E Yorke JA Ott E Local low dimensionality of atmospheric dynamics Phys Rev Lett 2001 86 5878 5881 10.1103/PhysRevLett.86.5878 11415384

[B24] Pérez-García VM Calvo GF Belmonte-Beitia J Diego D Pérez-Romasanta L Bright solitary waves in malignant gliomas Phys Rev E 2011 84 021921 10.1103/PhysRevE.84.021921 21929033

[B25] Hoffman RN Ponte RM Kostelich EJ Blumberg A Szunyogh I Vinogradov SV Henderson JM A simulation study using a local ensemble transform Kalman filter for data assimilation in New York Harbor J Atmos Ocean Tech 2008 25 1638 1656 10.1175/2008JTECHO565.1

[B26] Hoffman MJ Greybush SJ Wilson RJ Gyarmati G Hoffman RN Kalnay E Ide K Kostelich EJ Miyoshi T Szunyogh I An ensemble Kalman filter data assimilation system for the Martian atmosphere: Implementation and simulation experiments Icarus 2010 209 470 481 10.1016/j.icarus.2010.03.034

[B27] Wang X Bishop CH Julier SJ Which is better, an ensemble of positive-negative pairs or a centered spherical simplex ensemble? Mon Wea Rev 2004 132 1590 1605 10.1175/1520-0493(2004)132<1590:WIBAEO>2.0.CO;2

[B28] Beliën ATJ Paganetti PA Schwab ME Membrane-type 1 matrix metalloprotease (MT1-MMP) enables invasive migration of glioma cells in central nervous system white matter J Cell Biol 1999 144 373 384 10.1083/jcb.144.2.373 9922462 PMC2132902

[B29] Beadle C Assanah MC Monzo P Vallee R Rosenfeld SS Canoli P The role of myosin II in glioma invasion of the brain Mol Biol Cell 2008 19 3357 3368 10.1091/mbc.E08-03-0319 18495866 PMC2488307

[B30] Montana V Sontheimer H Bradykinin promotes the chemotactic invasion of primary brain tumor J Neurosci 2011 31 4858 4867 10.1523/JNEUROSCI.3825-10.2011 21451024 PMC3096850

[B31] Edelman LB Eddy JA Price ND *In silico *models of cancer WIREs Syst Biol Med 2010 2 438 459 10.1002/wsbm.75 PMC3157287 20836040

[B32] Deisboeck TS Zhang L Yoon J Costa J *In silico *cancer modeling: Is it ready for prime time? Nat Rev Clin Oncol 2009 6 34 42 10.1038/ncponc1237 PMC3212937 18852721

[B33] Bearer EL Lowengrub JS Frieboes HB Chuang YL Jin F Mand Ferrari Wise MS Agus DB Cristini V Multiparameter computational modeling of tumor invasion Cancer Res 2009 69 4493 4501 10.1158/0008-5472.CAN-08-3834 19366801 PMC2835777

[B34] Tsai MH Cook JA Chandramouli CV DeGraff W Yan H Zhao S Coleman CN Mitchell JB Chuang EY Gene expression profiling of breast, prostate, and glioma cells following single versus fractionated doses of radiation Cancer Res 2007 67 3845 3852 10.1158/0008-5472.CAN-06-4250 17440099

[B35] Tracqui P Cruywagen GC Woodward DE Bartoo GT Murray JD Alvord ECJ A mathematical model of glioma growth: the effect of chemotherapy on spatio-temporal growth Cell Prolif 1995 28 17 31 10.1111/j.1365-2184.1995.tb00036.x 7833383

[B36] Piette C Deprez M Roger T Noël A Foidart JM Munaut C The dexamethasone-induced inhibition of proliferation, migration, and invasion in glioma cell lines is antagonized by macrophage migration inhibitory factor (MIF) and can be enhanced by specific MIF inhibitors J Biol Chem 2009 284 32483 32492 10.1074/jbc.M109.014589 19759012 PMC2781663

[B37] Roniotis A Marias K Sakkalis V Zervakis M Diffusive modeling of glioma evolution: A review J Biomed Sci Engr 2010 3 501 508 10.4236/jbise.2010.35070

[B38] Hogea C Davatzikos C Biros G Modeling glioma growth and mass effect in 3D MR images of the brain Med Image Comput Comput Assist Interv 2007 10 642 650 18051113 10.1007/978-3-540-75757-3_78

[B39] Clatz O Sermesant M Bondiau PY Delingette H Warfield SK Malandian G Ayache N Realistic simulation of the 3d growth of brain tumors in MR images coupling diffusion with biomechanical deformation IEEE Trans Med Imaging 2005 24 1334 1346 16229419 10.1109/TMI.2005.857217 PMC2496876

[B40] Baek SJ Hunt BR Kalnay E Ott E Szunyogh I Local ensemble Kalman filtering in the presence of model bias Tellus A 2006 58 293 306

[B41] Zupanski D Zupanski M Model error estimation employing an ensemble data assimilation approach Mon Wea Rev 2006 134 1337 1354 10.1175/MWR3125.1

[B42] Orrell D Ensemble forecasting in a system with model error J Atmos Sci 2005 62 1652 1659 10.1175/JAS3406.1

[B43] Emblem KE Zoellner FG Tennoe B Nedregaard B Nome T Due-Tonnesson P Hald JK Schreie D Bjornerud A Predictive modeling in glioma grading from MR perfusion images using support vector machines Magn Reson Med 2008 60 945 952 10.1002/mrm.21736 18816815

[B44] Vannucci RC Barron TF Lerro D Antón SC Vannucii SJ Craniometric measures during development using MRI Neuroimage 2011 56 1855 1864 10.1016/j.neuroimage.2011.03.044 21439387

[B45] Ruoslahti E Brain extracellular matrix Glycobiology 1996 6 489 492 10.1093/glycob/6.5.489 8877368

[B46] Bonneh-Barkay D Wiley CA Brain extracellular matrix in neurodegeneration Brain Pathol 2009 19 573 585 10.1111/j.1750-3639.2008.00195.x 18662234 PMC2742568

[B47] Bauer R Ratzinger S Wales L Bosserhoff A Senner V Grifka J Grässel S Inhibition of collagen XVI expression reduces glioma cell invasiveness Cell Physiol Biochem 2011 27 217 226 10.1159/000327947 21471710

[B48] Rong Y Durden DL Van Meir EG Brat DJ "Pseudopalisading" necrosis in glioblastoma: A familiar morphologic feature that links vascular pathology, hypoxia, and angiogenesis J Neuropathol Exp Neurol 2006 65 529 539 10.1097/00005072-200606000-00001 16783163

